# The Extraction, Characterization and Biological Activity of Natural Products

**DOI:** 10.3390/plants12193382

**Published:** 2023-09-25

**Authors:** Linlin Yan, Aleksandra Cvetanović Kljakić

**Affiliations:** 1 Institute of Chemical Industry of Forest Products, CAF, Nanjing 210042, China; yanlinlin155@163.com; 2 Faculty of Technology, University of Novi Sad, Bulevar Cara Lazara 1, 21000 Novi Sad, Serbia

The aim of this Special Issue, entitled “The Extraction, Characterization and Biological Activity of Natural Products”, is to expand our knowledge and promote a better understanding of the latest scientific advances in the field of the extraction, isolation and characterization of biologically active compounds of natural plants. This Special Issue comprises 11 scientific papers (eight articles, one communication and two reviews), which provide insights into modern extraction techniques, new green extraction solvents, a novel analytical technique, the valorization of plants or bio-wastes, and the potential bioactive effects of plant or bio-waste materials as an alternative source of bioactive compounds. This summary aims to provide a concise overview of the valuable papers in this Special Issue, with the goal of enhancing their impact and citation rates. 

Natural products of plant origin have been widely used in various branches of industry, especially in the pharmaceutical, cosmetic and food industries. The interest in natural biomolecules arises from their lower incidence of side effects compared to synthetic compounds and the growing recognition by modern science of the significance of synergistic interactions between the biomolecules present in natural mixtures. The need for new products with increased functionality and improved characteristics that can meet the increasingly demanding requirements of consumers has developed. This requires intensive research in the field of bioactive molecules, and thus research regarding the isolation mechanisms has intensified. Therefore, more and more research is being conducted with the aim of in vitro and in vivo determination of their bio-activity, as well as elucidating their action mechanism. In addition to biological characterization, their chemical characterization is undoubtedly crucial, and research is intensifying in the field of development of analytical methods for their reliable detection, identification and quantification. Furthermore, it is very important to determine the potential toxicity of natural bioactive compounds, as well as the toxic doses, but also their metabolism in a living organism.

This Special Issue has published several articles pertaining to the extraction, characterization, and bio-activity evaluation of natural products derived from various plants. In one communication, Kim et al. (2022) [1] employed LC-QTOF-MS to characterize the bio-active constituents of *Dryopteris* sp. extracts from two Korean species (*Dryopteris lacera* and *Dryopteris bissetiana*). A variety of biological phenolic compounds were identified, including juglanin, 6-hydroxyluteolin 7-O-laminaribioside, peltatoside, kaempferitrin, hyperoside, astragalin, and neochlorogenic acid. Both *Dryopteris* sp. extracts exhibited potent antibacterial activity against Gram-positive pathogens, but not against Gram-negative bacteria. Salama et al. (2022) [2] identified 17 components by a GC-MS analysis of MeOH extracts from *Reichardia tingitana* aerial parts in Egypt. This study also indicated that *Reichardia*. tingitana shoots possess advantageous biological characteristics, including larvicidal effectiveness against A. aegypti, the dengue virus vector, as well as antioxidant, antibacterial, and cytotoxic properties. Another Egyptian researcher, El-Seadawy et al. (2022) [3], investigated the in vitro toxoplasmocidal and cytotoxic activities of different fractions extracted from *Cycas rumphii* Miq. leaves. Their findings showed that the ethyl acetate fraction exhibits potent toxoplasmocidal and cytotoxic activities against various cell lines, and seven compounds have been identified from this, including six known compounds and one new compound (bioflavonoid-di-C-glucoside) first reported in *Cycas rumphii* Miq. Leaves. Sookkhee et al. (2022) [4] investigate the antibacterial activity of ethanolic *Kaempferia parviflora* extracts and the combined effects of its specific compounds with gentamicin against clinical strains of carbapenem-resistant Klebsiella pneumoniae, Pseudomonas aeruginosa, and Acinetobacter baumannii. They found that the compound of 3,5,7,3′-4′-pentamethoxyflavone extracted from Kaempferia parviflora potentiated a synergistic effect with gentamicin against bacterial resistance in the tested bacteria. This research provides novel insights into the synergistic effects of antibiotics and herbal extracts for treating infections. Slighoua et al. (2022) [5] have characterized the phytochemical composition of hydro-ethanolic (HE) extracts from *Lavandula officinalis* Chaix, and evaluated the analgesic and wound-healing effects of both HE and polyphenolic (LOP) extracts through in vivo rat experiments and molecular docking simulations. LC-MS/MS analysis identified several phenolic compounds, including apigenin, catechin, and myricetin, and the GC-MS analysis revealed the presence of 19 volatile compounds, with triazole, D-glucose, hydroxyphenyl, and D-Ribofuranose as the major compounds. Moreover, both extracts showed high healing percentages, i.e., 99.31 and 92.88%, compared to the control groups, respectively. Molecular docking analysis showed that myricetin, amentoflavone, apigenin, and catechin exhibit the highest activity against the three enzyme receptors involved in wound-healing and analgesic. This study indicated that the potential of *L. officinalis* Chaix as a natural product source for pharmaceutical applications in analgesia and the promotion of burn-healing activity. Chamomile is a highly utilized medicinal plant worldwide, with extensive applications in both traditional and modern medicine. Cvetanović Kljakić et al. (2023) [6] employed an artificial neural network (ANN) model to optimize the parameters of the green extraction process to obtain phenolic extracts with a high content of required components. Under the optimized conditions (400 W, 30 min, and 1:80), the Chamomile extract exhibited a high content of total phenols (55.21 mg CAE/g) and total flavonoids (44.98 mg/g). LC-MS analysis confirmed the presence of 67 polyphenolic compounds, among which apigenin and apigenin-7-O-glucoside were dominant. In addition, in vitro evaluation experiments demonstrated that the optimized extract possessed potent antioxidant and glucosidase inhibitory activities. 

Some traditional medicine plants or herbs are widely recognized for their benefits in disease prevention and treatment, but they also present challenges, such as reduced efficacy and limited oral absorption. Fortunately, the development of medicinal plant-derived nanoparticles presents tremendous opportunity to improve therapeutic outcomes. Based on this, Soliman et al. (2023) [7] synthesized silver nanoparticles through a green approach utilizing Latex of Cynanchum acutum L. The resulting silver nanoparticles (Cy-AgNPs) were subsequently characterized by GC-MS, SEM, FT-IR and UV. Additionally, the genotoxicity and cytotoxicity effects of crude latex and various concentrations of Cy-AgNPs were investigated using Vicia faba as a model test plant. Natural extracts possess antibacterial properties and have the potential to replace preservatives in cosmetics while maintaining safety. Langová et al. (2023) [8] suggested that the hydroalcoholic extract of Achyrocline satureioides could be a natural and hypoallergenic alternative, with antimicrobial properties, for commonly used preservatives in cosmetic emulsions. The fruits of the large cranberry (*Vaccinium macrocarpon* Aiton) are a valuable source of biologically active compounds, such as anthocyanins, phenolic compounds, flavonols, et al. [9]. Šedbare et al. (2023) [9] indicated that freeze-dried cranberry fruit powder presents a promising source of anthocyanin-rich dietary supplements, and the incorporation of chitosan as an excipient in capsule formulations may offer a viable solution, enhancing anthocyanin stability and achieving modified release within the gastrointestinal tract.

Two reviews were published in our Special Issue, one of which systematically describes the application of metabolomics and network pharmacology as tools for exploring multi-target therapeutic approaches for traditional medicinal plants [10]. Based on a comprehensive analysis of data collected since 2000 from a variety of electronic scientific databases, this review explores the multi-target therapeutic approaches of Indian traditional medicinal plants in the treatment of various acute and chronic diseases. Another systematic review presents a comprehensive organization and summary of the significant research advances achieved through the utilization of alcoholic extracts from 14 Viscum species [11]. It thoroughly discusses and highlights various approaches applied to the genus Viscum, elucidates the targets and mechanisms of action of these alcoholic extracts, and provides guidance for future research and potential clinical applications. 

The papers published in this Special Issue delineate the extraction techniques, methods for identification and analysis, and chemical compositions of various plant extracts, as well as their bioactive properties, including their antioxidant, antibacterial, and larvicidal activity. 

## Data Availability

Not applicable.

## References

[1] Kim Y., Lim D.J., Song J.-S., Kim J.-A., Lee B.-H., Son Y.K. (2022). Identification and Comparison of Bioactive Components of Two *Dryopteris* sp. Extract Using LC-QTOF-MS. Plants.

[2] Salama S.A., Al-Faifi Z.E., El-Amier Y.A. (2022). Chemical Composition of *Reichardia tingitana* Methanolic Extract and Its Potential Antioxidant, Antimicrobial, Cytotoxic and Larvicidal Activity. Plants.

[3] El-Seadawy H.M., Abo El-Seoud K.A., El-Aasr M., Tawfik H.O., Ragab A.E. (2022). Toxoplasmocidal and Cytotoxic Activities Guided Isolation and Characterization of an Undescribed Bioflavonoid-di-C-glucoside from *Cycas rumphii* Miq. Cultivated in Egypt. Plants.

[4] Sookkhee S., Sakonwasun C., Mungkornasawakul P., Khamnoi P., Wikan N., Nimlamool W. (2022). Synergistic Effects of Some Methoxyflavones Extracted from Rhizome of *Kaempferia parviflora* Combined with Gentamicin against Carbapenem-Resistant Strains of *Klebsiella pneumoniae*, *Pseudomonas aeruginosa*, and *Acinetobacter baumannii*. Plants.

[5] Slighoua M., Chebaibi M., Mahdi I., Amrati F.E.-z., Conte R., Cordero M.A.W., Alotaibi A., Saghrouchni H., Agour A., Zair T. (2022). The LC-MS/MS Identification and Analgesic and Wound Healing Activities of *Lavandula officinalis* Chaix: In Vivo and In Silico Approaches. Plants.

[6] Cvetanović Kljakić A., Radosavljević M., Zengin G., Yan L., Gašić U., Kojić P., Torbica A., Belović M., Zeković Z. (2023). New Biological and Chemical Insights into Optimization of Chamomile Extracts by Using Artificial Neural Network (ANN) Model. Plants.

[7] Soliman M.I., Mohammed N.S., EL-Sherbeny G., Safhi F.A., ALshamrani S.M., Alyamani A.A., Alharthi B., Qahl S.H., Al Kashgry N.A.T., Abd-Ellatif S. (2023). Antibacterial, Antioxidant Activities, GC-Mass Characterization, and Cyto/Genotoxicity Effect of Green Synthesis of Silver Nanoparticles Using Latex of *Cynanchum acutum* L.. Plants.

[8] Langová D., Córdoba M.A.M., Sorrechia R., Hoová J., Svoboda Z., Mikulíková R., Correa M.A., Pietro R.C.L.R., Márová I. (2023). *Achyrocline satureioides* Hydroalcoholic Extract as a Hypoallergenic Antimicrobial Substitute of Natural Origin for Commonly Used Preservatives in Cosmetic Emulsions. Plants.

[9] Šedbare R., Janulis V., Ramanauskiene K. (2023). Formulation and Biopharmaceutical Evaluation of Capsules Containing Freeze-Dried Cranberry Fruit Powder. Plants.

[10] Sharma B., Yadav D.K. (2022). Metabolomics and Network Pharmacology in the Exploration of the Multi-Targeted Therapeutic Approach of Traditional Medicinal Plants. Plants.

[11] Melo M.N.d.O., Batista J.V.d.C., Peñaloza E.M.C., Oliveira A.P., Garrett R., Baumgartner S., Holandino C. (2023). A Scoping Review of Genus Viscum: Biological and Chemical Aspects of Alcoholic Extracts. Plants.

